# Persistent elevation of intrathecal pro-inflammatory cytokines leads to multiple sclerosis-like cortical demyelination and neurodegeneration

**DOI:** 10.1186/s40478-020-00938-1

**Published:** 2020-05-12

**Authors:** Rachel E. James, Renee Schalks, Eleanor Browne, Ioanna Eleftheriadou, Carmen Picon Munoz, Nicholas D. Mazarakis, Richard Reynolds

**Affiliations:** 1 Division of Neuroscience, Department of Brain Sciences, Imperial College London, Hammersmith Hospital Campus, Burlington Danes Building, Du Cane Road, London, W12 0NN UK; 2 Centre for Molecular Neuropathology, LKC School of Medicine, Nanyang Technological University, Singapore, Singapore

**Keywords:** Multiple sclerosis, Neurodegeneration, Demyelination, Meninges, Inflammation, Animal model

## Abstract

Analysis of isolated meninges and cerebrospinal fluid (CSF) of post-mortem MS cases has shown increased gene and protein expression for the pro-inflammatory cytokines: tumour necrosis factor (TNF) and interferon-γ (IFNγ). Here we tested the hypothesis that persistent production of these cytokines in the meningeal compartment and diffusion into underlying GM can drive chronic MS-like GM pathology. Lentiviral transfer vectors were injected into the sagittal sulcus of DA rats to deliver continuous expression of TNF + IFNγ transgenes in the meninges and the resulting neuropathology analysed after 1 and 2 months. Injection of TNF + IFNγ viral vectors, with or without prior MOG immunisation, induced extensive immune cell infiltration (CD4+ and CD8+ T-cells, CD79a + B-cells and macrophages) in the meninges by 28 dpi, which remained at 2 months. Control GFP viral vector did not induce infiltration. Subpial demyelination was seen underlying these infiltrates, which was partly dependant on prior myelin oligodendrocyte glycoprotein (MOG) immunisation. A significant decrease in neuronal numbers was seen at 28 and 56 days in cortical layers II-V that was independent of MOG immunisation. RNA analysis at 28 dpi showed an increase in expression of necroptotic pathway genes, including RIP3, MLKL, cIAP2 and Nox2. PhosphoRIP3+ and phosphoMLKL+ neurons were present in TNF + IFNγ vector injected animals, indicating activation of necroptosis. Our results suggest that persistent expression of TNF in the presence of IFNγ is a potent inducer of meningeal inflammation and can activate TNF signalling pathways in cortical cells leading to neuronal death and subpial demyelination and thus may contribute to clinical progression in MS.

## Introduction

The progressive phase of multiple sclerosis (MS) is characterised by an increasing burden of white matter demyelination, and axon loss and grey matter (GM) pathology that together contribute to irreversible accumulation of motor, sensory and cognitive symptoms [6, 29]. It is now widely thought that accumulating axon and neuronal loss are the main pathological correlates of clinical progression [6, 29, 31]. Although there is substantial evidence supporting an inflammatory basis to the neurodegenerative pathology [13, 15, 23, 28], the molecular mechanisms are unresolved. We have previously demonstrated that the extent of immune infiltration in the cerebral meninges correlates with the degree of GM demyelination, microglial activation, axonal pathology and neuronal loss [17, 22, 23]. A gradient of neuronal loss within the motor cortex was associated with a shorter time to milestones of clinical progression, such as shorter time to wheelchair use, shorter disease duration and younger age at death [17, 23, 29]. Further studies have demonstrated that meningeal inflammation and associated sub-pial demyelination and neuronal loss can occur at the early stages of MS [3, 21].

In secondary progressive MS (SPMS) post-mortem brains, greater leptomeningeal inflammation and grey matter demyelination were associated with elevated CSF protein levels of the proinflammatory cytokines TNF and IFNγ and the B-lymphocyte chemokine CXCL13 [24]. Moreover, isolated meninges from the post-mortem MS brains that exhibited increased meningeal inflammation showed increased gene expression for TNF, IFNγ and CXCL13. Increased levels of TNF, IFNγ and CXCL13 in the CSF at MS diagnosis also defined those patients with the most extensive cortical GM damage on MRI [24]. CXCL12, LIGHT, IL6 and IL10 were also suggested to play a significant but smaller role. These data imply that meningeal immune infiltrates may release inflammatory mediators, such as TNF and IFNγ, that diffuse through the underlying cerebral cortex to, directly and/or indirectly, cause demyelination and neurodegeneration leading to general neurological decline and cognitive dysfunction [17, 23]. Other studies have demonstrated that both neurodegeneration and meningeal inflammation can be widespread in the MS brain [7, 10, 15]. Therefore, it seems likely that the extent of these pathologies could at least in part explain the degree of motor, sensory and cognitive deficits seen in individual patients. However, these observations in human tissue need to be confirmed using an experimental approach to demonstrate that the persistently elevated levels of intrathecal proinflammatory cytokines seen in MS can indeed lead to cortical pathology, including neuronal loss. Acute studies involving injection of recombinant TNF and IFNγ into the rat cortical parenchyma [26, 35] or subarachnoid space [14] gave rise to a reversible subpial demyelinating pathology without neuronal loss, suggesting the likely importance of these two pro-inflammatory cytokines.

Here we have asked whether chronic demyelination and accumulating neuronal loss can result from persistently elevated levels of TNF and IFNγ in the subarachnoid space of the rat brain. In addition, we have investigated the contribution of anti-myelin autoimmunity to these pathologies in light of the fact that subpial demyelination in a previous acute model [14] only occurred after immunisation with MOG. Establishing a causal relationship between these intrathecal cytokines, chronic meningeal inflammation, cortical pathology and increasing disability will open up an avenue for the development of an effective approach to slowing the progressive course of MS.

## Material and methods

### Viral vector production

For lentiviral production we utilised a HIV-1 transfer plasmid (pRRL-sincppt-CMV-eGFP-WPRE genome plasmid) that carries the human cytomegalovirus promoter (CMV). Complementary DNA sequences for human tumour necrosis factor (TNF) or interferon gamma (IFNƴ) were codon optimised for rat (see Additional file 1A) including a 5′ Kozak sequence and synthesised by Gene Art with Xba1 and Sal1 restriction sites (Life Sciences, Paisley, UK). The human transgene DNA fragments were excised by restriction digest with Xba1 and Sal1 before purification using preparative agarose gel electrophoresis. The eGFP was removed from the transfer plasmid by digestion with Xba1 and Sal1 and the transgene DNA fragments ligated in-frame using the same restriction sites.

Recombinant HIV-1 based lentiviruses were produced using four plasmid co-transfection of HEK-293 T cells as described previously [8]. Briefly, HEK-293 T cells were transfected with 15 μg vector plasmid (pRRLsincppt-CMV-TNF-WPRE, pRRLsincppt-CMV-IFNƴ -WPRE or pRRLsincppt-CMV-GFP-WPRE), 15 μg of the packaging vector plasmids expressing the HIV-1 gag/pol gene (pMD2-LgRRE), 3 μg of HIV-1 Rev. (pRSV-Rev) and 5.1 μg VSV-G envelope plasmids (pMD2-VSV-G) following the addition of 2 mol/L CaCl_2_. Lentivirus was concentrated from supernatant using ultracentrifugation and the genome copy number was calculated using the Clontech Lenti-X qRT-PCR Titration kit (Takara).

### Transduction of primary meningeal cultures

Primary meningeal cultures were prepared from P4 Sprague Dawley pups. Briefly, the meninges were dissected from the surface of the cortex and digested with papain and DNAse. Cells were plated on poly-l-lysine coated 8-well chamber slides or 24 well plates (Corning) and grown in DMEM/F12 (Sigma, UK) supplemented with 10% calf serum, 1% Penicillin/Streptomycin and 1% L-Glutamine (Sigma, UK). After 1 week in culture cells were transduced at MOI 50 with LV-TNF, LV-IFNγ or LV-GFP and the supernatant harvested after 24 or 48 h (see Additional file 1B). Enzyme-linked immunosorbent assay for human TNF or IFNγ was performed in triplicate using the DUO set ELISA kit (DY210, TNF; DY285 IFNγ, R&D Systems, Abingdon, UK) (see Additional file 1C).

### Animals

Eight to 10 week old female Dark Agouti (DA) rats (140-160 g) were obtained from Janvier (France) and kept in groups of four in a 12 h light/dark cycle with food and water provided ad libitum. All animal experiments were carried out under approval from the UK Home Office. For all groups n was 5 to 7 animals with a total of 68 animals for all experiments. Animals were randomly assigned to groups.

### Induction of sub-clinical experimental autoimmune encephalomyelitis

Sub-clinical MOG-induced EAE was induced as described previously [14]. Rats were anaesthetised with isofluorane and immunised intradermally at the base of the tail (dorsal aspect) with 5 μg recombinant mouse MOG (amino acids 1–119, corresponding to the external Ig-like domain) diluted in sterile PBS and emulsified in an equal volume of incomplete Freund’s adjuvant (IFA, Sigma, 100 μl total volume) or with PBS alone emulsified in IFA. It has been reported that IFA can reduce the clinical severity of autoimmune models by preventing T cell proliferation and reducing expression of cytokines, chemokines, and chemokine receptors on CNS-infiltrating mononuclear cells [42]. Therefore, we have used IFA/PBS as an additional control to the naïve animals for comparisons to the subclinical EAE group induced with IFA/MOG. Naïve animals received no treatment. Rats exhibited no clinical signs of EAE following low dose MOG immunisation [14]. Production of peripheral anti-MOG IgG1 and IgG2a antibody titres in MOG immunised animals was confirmed using ELISA (see Additional file 2). Antibodies to MOG were not present in IFA immunised animals.

### Intracerebral injection of viral vectors

Rats were either used naïve with no treatment, immunised with MOG in IFA or IFA alone and stereotactic surgery performed at 20–23 days post-immunisation under isofluorane anaesthesia [14]. A 5 mm hole was drilled in the skull in the midline 0.9 mm caudal to bregma. A finely calibrated glass capillary attached to a 26S fixed needle 10 μl Hamilton syringe was inserted to a depth of 2.3 mm below the dural surface. The rats were then injected with 4 μl of lentiviral mixture diluted in TSSM with 0.5 mM monastral blue tracer at a rate of 0.20 μl/min over a 20 min period. Viruses were injected at a total of 5 × 10^8^ genomic copies (GC) for TNF and GFP and 5 × 10^7^ GCs for IFNγ. The needle was left in place for 10 min to allow diffusion of the sample from the area of injection and then slowly withdrawn.

### Catwalk XT automated gait analysis system

Quantitative fully automated gait analysis was conducted with the CatWalk system (Catwalk XT, Noldus Information Technology, Netherlands). For the detection of all parameters the camera gain was set to 20 (dB) and the detection threshold to 0.1. For each animal and analysed time point, 6 compliant runs with a run duration of 0.5–5 s and maximum allowed speed variation of 60%, were acquired per trial.

### Tissue processing

Rats were killed by intraperitoneal injection of 200 mg/kg of sodium pentobarbitone and a CSF sample taken from the cisterna magna using a 26G needle (Hamilton) before cessation of breathing, with the animal on a stereotaxic frame. Animals were perfused through the left ventricle with PBS followed by 4% paraformaldehyde at 28 and 56 days post viral injection and tissue prepared as described previously [14].

### Tissue and CSF analysis

Protein expression from the injected transgenes was measured in snap-frozen rat CSF on electrochemiluminescence (ECL) Proinflammatory panel 1 human assay plates (Meso Scale Discovery, USA) as described previously [24].

Cortical tissue for each animal was dissected from 4 × 10 μm sections mounted on RNase free slides using a scalpel, taking extra care not to include meningeal tissue. RNA was extracted using the FFPE mini tissue kit (Qiagen) and quantified using a Nandrop ND1000 spectrophotometer. 700 ng RNA was reverse transcribed for each sample using RT2 First Strand Kit before a proprietary amplification step using the RT2 PreAMP cDNA synthesis kit for the Rat necrosis PCR array (Qiagen, PBM-141-Z). The PreAMP mix was run on RT2 profiler PCR rat necrosis array plates (Qiagen, PARN-141ZA-2) on a Stratagene MX3005P system. Relative fold changes were calculated using SABiosciences web-based PCR array data analysis tool.

For RT-PCR experiments 200 ng of RNA was reverse transcribed using nanoscript2 kit from Primerdesign. Real Time PCR was performed using the Stratagene MX3005P system as described previously [12]. Primers were custom designed by PrimerDesign (PrimerDesign, UK). TOP1 and YHMAZ were used as normalising genes. Relative expression was calculated using the delta delta CT method.

### Immunohistochemistry

Immunohistochemistry/immunofluorescence was performed as previously described [14] using monoclonal and polyclonal antibodies listed in Table 1. Tiled digital images were obtained at × 10 or × 20 magnification and coded for blinded analysis using ImageJ software. For each animal, immunofluorescence tiled images were acquired from 4 × 10 μm sections stained with anti-MOG antibody to quantify demyelination. Images were converted to 8-bit and automated Otsu thresholding was used to calculate the percentage area of regions of interest stained by the MOG antibody.

**Table 1:** Table of species, concentration and source of the antibodies used for immunostaining

CD4	T helper cells	Mouse	1:2000	AbD Serotec
CD8	Cytotoxic T cells	Mouse	1:2000	AbD Serotec
CD79a1	B cells	Mouse	1:2000	Thermoscientific
IFNγ	Cytokine	Mouse	1:100	Santa Cruz
TNF	Cytokine	Goat	1:100	R&D Systems
Iba1	Microglia/Macrophages	Rabbit	1:2000	Wako, Japan
NeuN	Neuronal Nuclei	Mouse	1:1000	Chemicon
MOG	Myelin/oligodendrocytes	Mouse	1:50	R. Reynolds
CNPase	Myelin/oligodendrocytes	Mouse	1:1000	Millipore
NFH	200 kDa Neurofilament protein	Mouse	1:1000	Chemicon
Cl-Casp3	Apoptotic cells	Rabbit	1:1000	Cell Signalling
MLKL	TNF signalling	Rabbit Mouse	1:500 1:250	Cell signalling Santa Cruz
pMLKL	Necroptotic cells	Rabbit	1:500	Abcam
RIPK3	TNF signalling	Rabbit	1:300	Sigma
pRIPK3	Necroptotic cells	Rabbit	1:500	Abcam
Olig2	Oligodendrocytes	Rabbit	1:500	Abcam

Meningeal lymphocyte numbers were counted manually from digital images of the CD4, CD8 and CD79a immunostained sections, using a region of interest that spanned from the base of the sagittal sulcus to the position of the lateral blood vessels visible in the meninges, and were expressed as total cells per region of interest. For both neuronal and microglial numbers, regions of interest were drawn that outlined the cortical layers or midline regions using ImageJ. Cortical layers were determined using co-staining with DAPI and Neun/HuCD to identify different cortical regions [14]. NeuN+ and Iba1+ cell numbers were counted manually at 20x magnification using the ImageJ cell counter tool. The total number of cells for all the counts was then divided by the total area of the region of interest to give the total cells/mm^2^. For lymphocytes, neurons and microglia, the number of cells was calculated from four sections per animal that were spaced 10 μm apart covering a total distance of 80 μm per animal.

### Statistical analysis

Graphpad Prism8 statistical software (La Jolla, CA, USA) was used in all cases to present the data and to conduct statistical analysis. All data on graphs are expressed as mean ± SEM except for PCR data which is shown with propagated standard error. Group comparisons for cell quantifications were analysed by one-way ANOVA with Tukey test for multiple comparisons. For Catwalk data we used two-way repeated measures ANOVA followed by Bonferroni’s post-hoc comparison tests when appropriate. A *P*-value of < 0.05 was considered significant in all cases.

## Results

### Expression of TNF and IFNγ transgenes

**Fig. 1:**
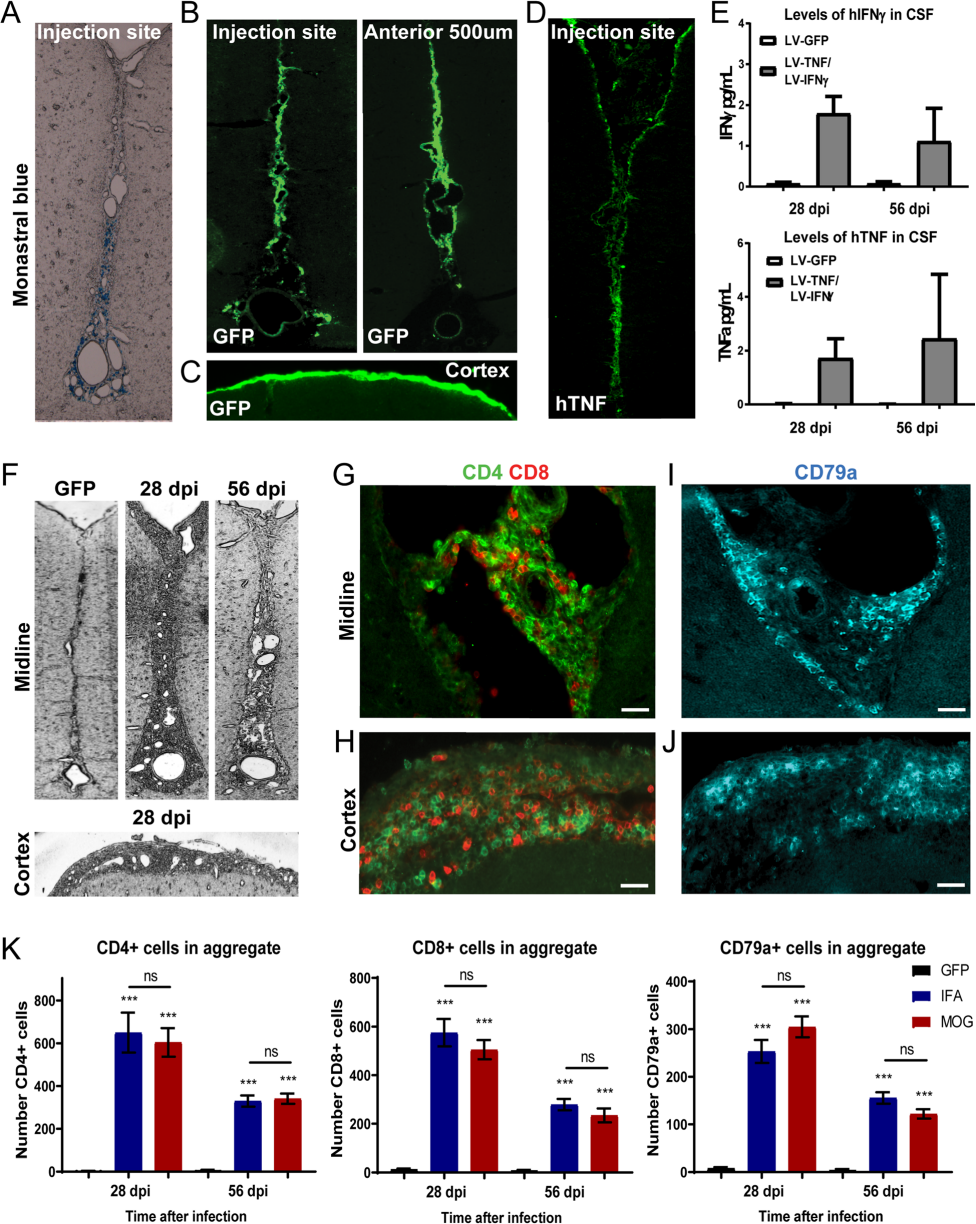
Injection of cytokine expressing vectors into the subarachnoid space of rats leads to meningeal inflammation. Lentiviral vectors (LV) expressing TNF and IFNγ were injected into the subarachnoid space where the dye monastral blue was used to identify the correct positioning and location of the injection site (**a**). Use of a LV expressing eGFP showed significant transduction of meningeal cells lining the cortex down the midline (**b**), spreading to at least 500 μm anterior to the injection site (**b**) and along the cortical surface (**c**). Immunostaining for human TNF showed that expression of the human TNF transgene occurred in cells along the length of the midline (**d**). Significant levels of human transgene derived TNF and IFNγ were detectable in CSF from cytokine LV but not from GFP LV injected animals at both 28 and 56 days post-injection (**e**). Low magnification brightfield images of MOG immunised animals showed that injection of vectors for TNF and IFNγ led to the formation of large dense infiltrates of cells within the sagittal sulcus and across the surface of the cortex at 28 days post-injection, which were still present at 56 days (**f**), whereas the control GFP vector did not induce inflammation. Immunofluorescent staining showed the extensive infiltration of CD4+ and CD8+ T cells in the meninges within the sagittal sulcus and overlying the cortex of cytokine vector injected MOG animals (**g,h**). CD79a + B-cell aggregates in serial sections of the same area appeared to form more discrete clusters (**i,j**). The maximal infiltration of T- and B-cells into the sagittal sulcus was seen 28 days after injection for both IFA and MOG immunised animals and decreased by approximately 50% by 56 days (**k**), with no significant difference between animals immunised with MOG or IFA (1-way analysis of variance with Tukey correction, *** *P* < 0.001 compared to GFP). CD4+ and CD8+ T cell numbers were approximately equal and twice that of B-cells. Data represents mean ± SEM from *n* = 5–6 per group. Scale bars = 20 μm

Primary meningeal cells could be 100% transduced with viral vectors for TNF, IFNγ or eGFP at an MOI of 50, demonstrating good tropism of the VSV-G envelope for meningeal cells (see Additional file 1B). Immunofluorescence using antibodies specific for human TNF and IFNγ showed high levels of cytoplasmic staining in transduced cells. Following stereotaxic injection into the subarachnoid space of the sagittal sulcus, as indicated by the presence of monastral blue dye (Fig. 1a), meningeal cells lining the sagittal sulcus were successfully transduced, as shown by the expression of GFP (Fig. 1b). Strong GFP expression could also be detected in meningeal cells lining the outer surface of the cortex (Fig. 1c). Immunofluorescence against human TNF showed expression of the human TNF transgene in transduced meningeal cells down the sagittal sulcus (Fig. 1d). Protein expression from the TNF and IFNγ transgenes was detected in CSF samples and maintained at both 28 and 56 days post injection (dpi), whilst levels were undetectable in GFP vector injected animals (Fig. 1e). This analysis was specific for the human transgene and did not cross react with the rat cytokines.

### Meningeal inflammation after TNF and IFNγ overexpression in the subarachnoid space

The formation of dense cellular infiltrates, down the entire depth of the sulcus and extending across the surface of the cortex, was observed following injection of TNF and IFNγ vectors into the subarachnoid space, at both 28 and 56 dpi (illustrated for MOG immunised animals in Fig. 1f), whereas injection of the control GFP vector did not result in infiltration. This meningeal inflammation was composed of both B and T cells (illustrated for MOG immunised animals in Fig. 1g-j) and the cells became densely packed, filling the entire subarachnoid space (Fig. 1g-j). No qualitative difference was seen between IFA only and IFA + MOG immunised animals. Whilst the CD4+ and CD8+ T-cells were roughly evenly distributed throughout the space (Fig. 1g,h), CD79a + B cells appeared to form more discrete clusters (Fig. 1i,j). While this is an expression pattern frequently seen in lymphoid-like aggregates in post-mortem MS brain [22], further work would be required to determine whether these aggregates possess any features of lymphoid-like tissues. Very few immune cells were seen in the underlying cortical parenchyma at any timepoint (not shown). Meningeal infiltration was greatest at 28 dpi compared to 56 dpi in the cytokine vector injected animals and showed no significant difference between MOG and IFA immunised animals at any timepoint (Fig. 1k), whilst lymphocyte numbers were negligible in GFP vector injected MOG immunised animals. The numbers of infiltrating CD4+ and CD8+ T-cells were approximately equal and twice the number of CD79a + B-cells in both IFA alone and IFA + MOG immunised cytokine vector injected animals (Fig. 1k).

### Demyelination and microglial activation

**Fig. 2:**
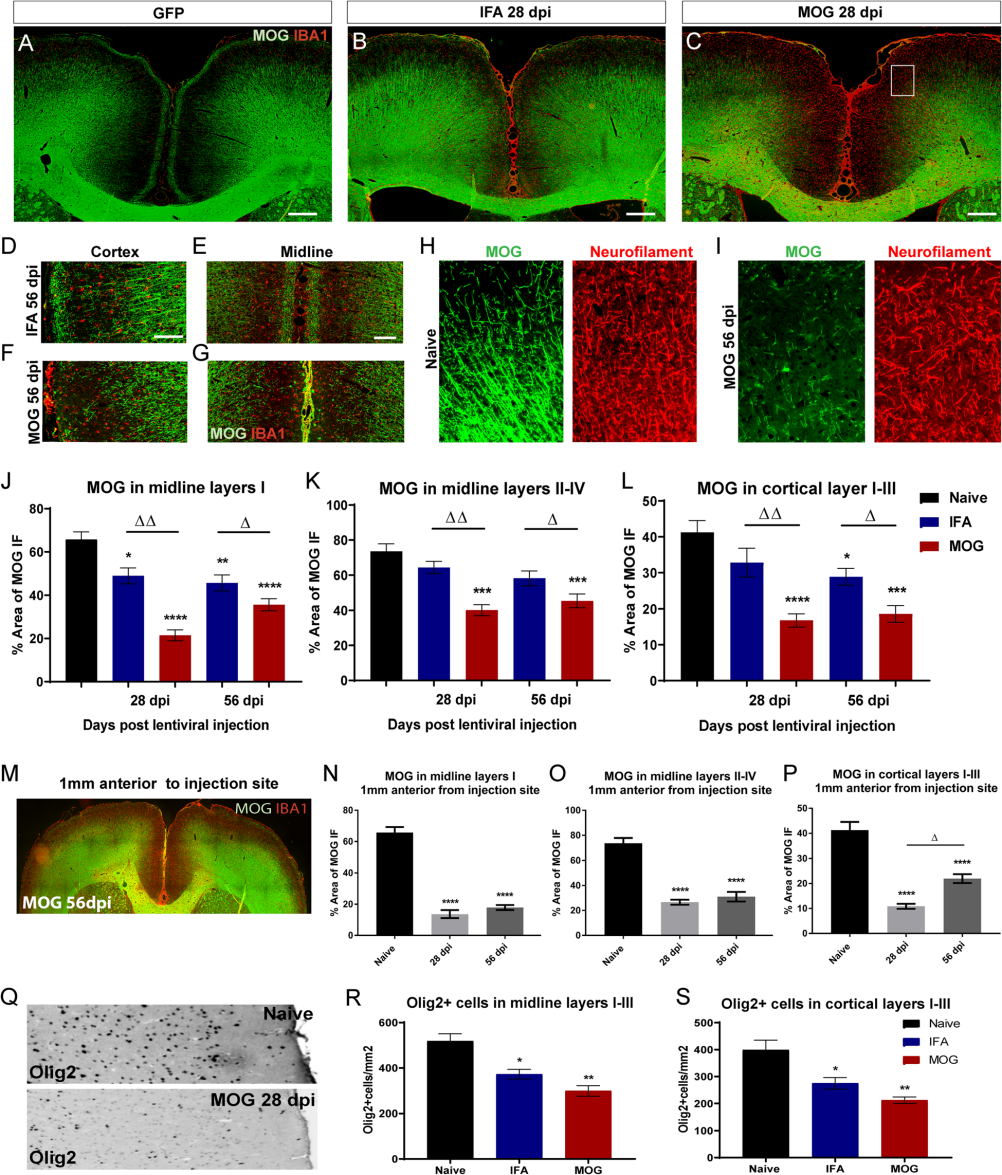
Chronic expression of cytokines leads to demyelination of subpial cortical layers. Immunofluorescence for MOG and IBA1 shows no demyelination in eGFP vector injected animals (**a**), but widespread demyelination in the outer cortical layers at 28 days after cytokine lentiviral injection in IFA (**b**) and MOG (**c**) immunised animals. A decrease of MOG staining can be seen in IFA animals in the cortical (**d**) and midline layers (**e**), which was greater at 56 days in MOG immunised animals (**f-g**). Areas with loss of MOG at 56 dpi (white box in C) showed similar levels of 200 kd neurofilament protein when compared to naive (**h-i**). The degree of demyelination was significantly different for both IFA and MOG immunised animals at 28 and 56 days in midline layer I (**j**), but only in MOG immunised animals in midline layer II-IV, compared to naïve (**k**)(1-way analysis of variance with Tukey post test, *P < 0.05, ***P < 0.001, ****P < 0.0001, cytokine vs naïve. Δ = *p* < 0.05, ΔΔ = *p* < 0.001 IFA vs MOG). Demyelination of cortical layers I-III was significant in MOG immunised animals at both 28 and 56 dpi, but only at 56 days in IFA animals (**l**). Subpial demyelination also extended in the rostral plane in MOG immunised animals at 56 days (**m**). Quantification of demyelination 1 mm anterior to the injection site in MOG immunised animals was equal to or greater than at the injection site at both 28 and 56 days after cytokine vector injection (**n-p**). Immunostaining for the transcription factor Olig2 revealed a substantial reduction in oligodendroglial lineage cells in cortical layers of MOG animals when compared to naïve (**q**), both for IFA and MOG immunised animals at 28 days post injection in the midline (**r**) and cortical layers (**s**), with no significant difference between IFA and MOG immunised animals (data are mean +/−SEM from *n* = 5–6 animals per group. Statistics: 1-way analysis of variance with Tukey post test. *p < 0.05, **P < 0.01). Scale bars = 300 μm (**a-c**), 50 μm (**d,e**)

No observable demyelination occurred in animals injected with GFP vector (Fig. 2a), irrespective of their immunisation status. Subpial demyelination of the upper cortical layers was observed in animals immunised with IFA alone and injected with cytokine vectors (illustrated at 28 days, Fig. 2b), which was even more extensive in IFA + MOG immunised and cytokine vector injected animals and reached to deeper levels of the cortex (illustrarted at 28 days, Fig. 2c). Demyelination extended from the corpus callosum boundary of the sagittal sulcus to the dorsal surface of the cortex and then into the subpial surface of the cortex laterally from the midline (Fig. 2b-g). This subpial demyelination was further confirmed with immunofluorescence for CNPase, a second myelin marker, which showed the same pattern of loss (see Additional file 2). Staining for neurofilament protein in layers II/III showed little difference when comparing areas of demyelination in MOG 56 dpi vector injected and naïve animals (Fig. 2h,i), indicating primary demyelination rather than secondary demyelination due to tissue necrosis. Quantification of the loss of MOG staining demonstrated that demyelination was greater in animals immunised with MOG compared to those immunised with IFA alone (Fig. 2j,k,l), both in the midline (15% in IFA versus 25% in MOG; Fig. 2j,k) and subpial cortical regions (12% in IFA versus 22% in MOG; Fig. 2l). At 56 dpi both IFA and MOG immunised animals continued to show significant levels of subpial demyelination in cortical layers compared to naïve animals (Fig. 2l). Subpial demyelination also extended in the rostral plane and showed the same pattern of midline and lateral cortical loss of MOG staining (Fig. 2m). Quantification of demyelination 1 mm anterior to the injection site in MOG immunised animals showed an extent of demyelination that was equal to, if not greater than, that seen at the injection site (Fig. 2n-p).

In order to quantify any effects on oligodendroglial lineage cells, we examined the presence of Olig2^+^ cells within the demyelinated regions of the cortex in cytokine vector injected animals at 28 dpi compared to naïve controls (Fig. 2q) and found a significant decrease in the number of Olig2^+^ cells in both IFA (33% decrease) and MOG immunised animals (47% decrease)(Fig. 2r,s).

**Fig. 3:**
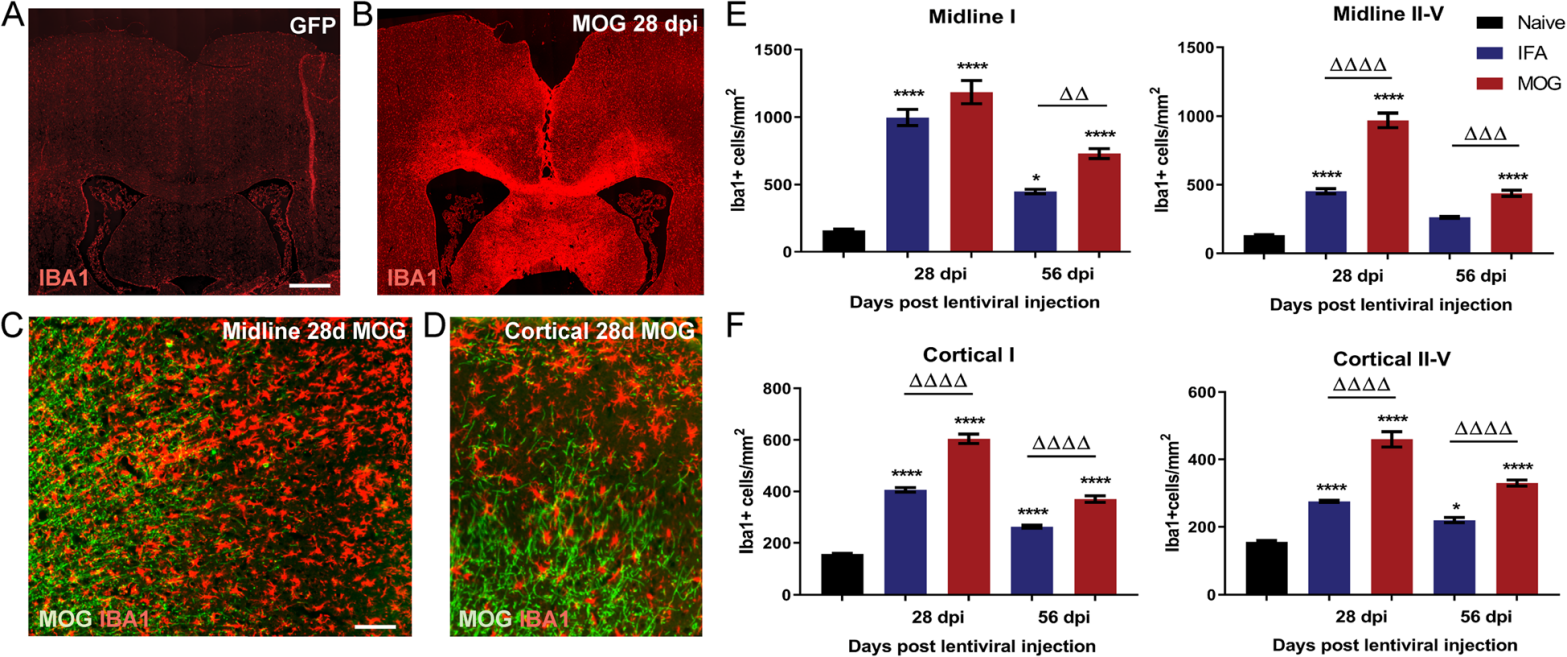
Viral delivery of cytokines into the CSF caused microglial activation in the underlying cortex. Microglial activation, indicated by IBA1 reactivity, was widespread in all cortical layers and the corpus callosum at 28 days (illustrated for MOG immunised animals) post cytokine viral vector injection, whereas injection with eGFP vector led to minimal Iba1 expression that was not different from naïve rats (**a,b**). Subpial demyelinated lesions in animals immunised with MOG and injected with cytokine vectors were characterised by large numbers of Iba1+ microglia with a highly activated morphology at 28 dpi (**c,d**). No amoeboid-like macrophages could be seen in the GM parenchyma at any stage. The number of Iba1+ cells was significantly increased in the cortical layers of both IFA and MOG immunised cytokine vector injected animals in all regions and at all timepoints, except for the midline II-IV at 56 dpi (**e,f**), with the greatest increases seen in MOG immunised animals. The greatest increase in Iba1+ cells was seen in the midline layer I region, closest to the immune aggregates in the sagittal sulcus (**e**), with slightly lower numbers in midline layer II-V (**e**). Iba1+ numbers were higher in cortical layer I than underlying layer II-V (**f**). Data are presented as mean ± SEM. Statistics: one-way ANOVA with Tukey post-hoc test. **** *P* < 0.0001, * *P* < 0.05 naïve versus cytokine or ΔΔΔΔ *P* < 0.0001. ΔΔΔ *P* < 0.001, ΔΔ *P* < 0.01, Δ *p* < 0.05 for comparison between 28 and 56 dpi for the same group (IFA vs IFA). Scale bar = 200 μm (**a,b**), 25 μm (**c,d**)

Microglial activation was widespread in cytokine vector injected animals at 28 days after injection, both IFA only and IFA + MOG immunised, when compared to GFP vector injected ones and occured throughout cortical layers I-VI and the corpus callosum (illustrated for MOG immunised animals in Fig. 3a,b), closely associated with demyelination in the midline and subpial cortical layers (Fig. 3c,d). Amoeboid macrophages were not seen in the areas of demyelination (Fig. 3c,d), similar to findings in the MS cortex [4, 23]. Microglial numbers in cytokine vector injected animals were increased at both 28 and 56 dpi when compared to naïve, but to a significantly greater extent in MOG-immunised versus IFA immunised animals (Fig. 3e,f) and were highest in parenchymal layers in close proximity to the sagittal sulcus (Fig. 3e) and the upper cortical layer I in proximity to meningeal immune cell infiltrates (Fig. 3f). No difference in microglial activation was seen in GFP vector injected control animals when compared to naives (not shown).

### Loss of neurons in subpial cortical layers

**Fig. 4:**
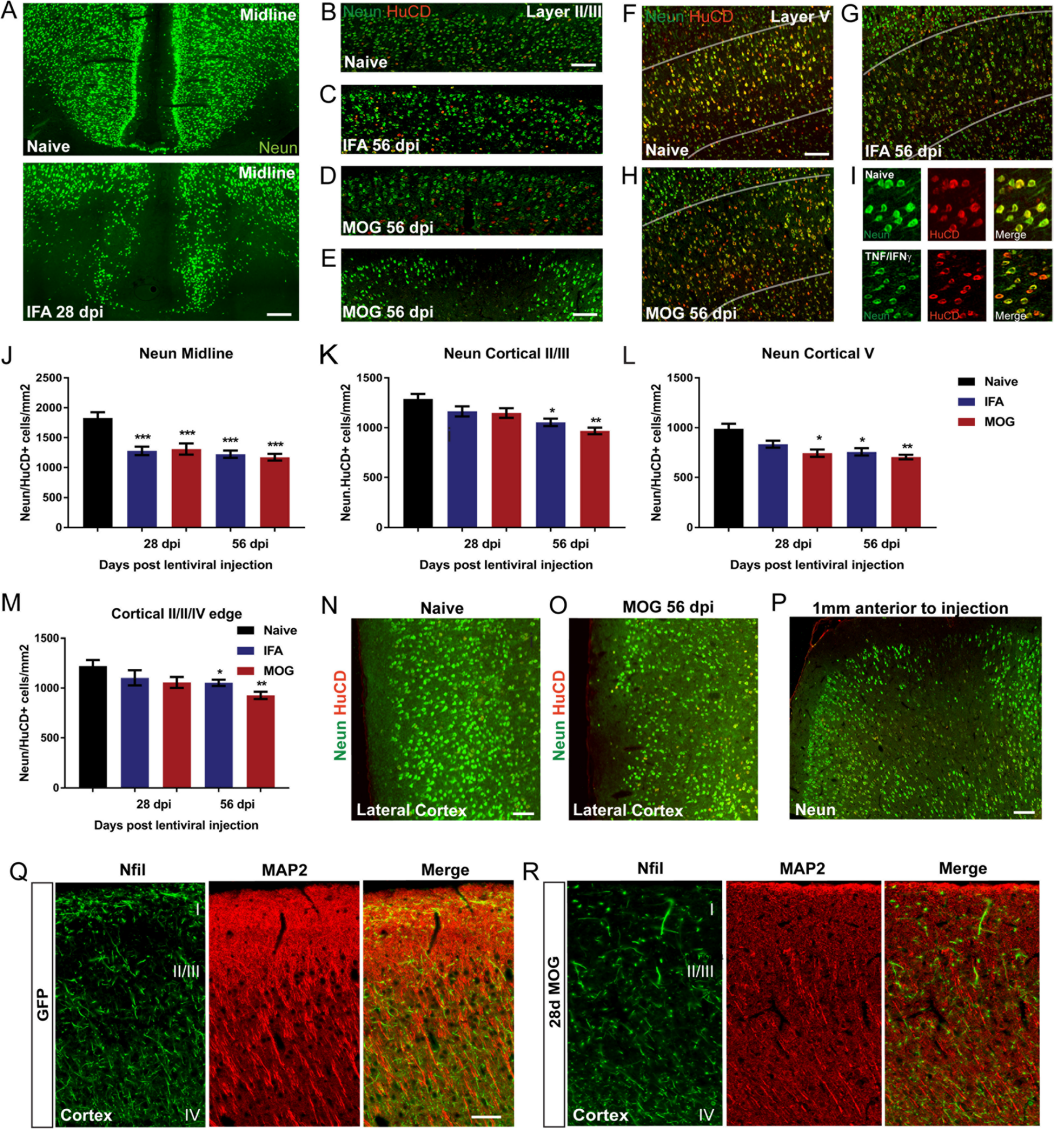
Neuronal loss in the cortical parenchyma. Loss of neuronal NeuN staining is seen in layer II along the sagittal sulcus and in layer V/VI bordering the corpus callosum at 28 days following cytokine vector injection (illustrated for IFA animals in **a**). No neuronal loss was seen in GFP vector animals (not shown). A decreased density of NeuN+/HuCD+ expressing neurons was seen at 56 days after cytokine vector injection in cortical layers II/III in both IFA (**c**) and MOG (**d,e**) immunised animals compared to naïve (**b**). Extensive regions spreading down from the pial surface in some animals displayed loss of NeuN/HuC/D+ cells (**e**). A reduction in NeuN+/HuCD+ neurons was present in cortical layer V (white lines) in IFA and MOG cytokine vector injected animals at 56 dpi (**f-h**). Images of co-staining with HuC/D and NeuN reveal the loss of NeuN expression in some neurons (**i**). Neuronal numbers were decreased in midline regions (layers I-IV) in both MOG and IFA immunised animals at 28 and 56 dpi (**j**). Neuronal loss in the upper layers II/III developed more gradually in both MOG and IFA animals and only became significant at 56 dpi (**k**). In layer V neuronal loss was significant at 28 and 56 dpi in MOG animals but only at 56 dpi in IFA animals (**l**). Levels of neuronal loss were similar for MOG or IFA animals at 56 dpi (**k,l**). Neuronal loss only became significant in the lateral cortex at 56 dpi (**m-o**). Neuronal loss could also be seen 1 mm anterior to the injection site (illustrated for a MOG animal at 56 dpi in **p**). Immunostaining for NFil and MAP2 in upper cortical layers revealed a reduction of staining in the apical dendritic tufts and the descending dendritic bundles that extended into layer II/III (illustrated for a MOG animal at 28 dpi in **q,r**). 1-way ANOVA with Tukey post multiple comparisons test, ***P < 0.001, **P < 0.01, *P < 0.05. Data represents mean ± SEM n = 5–7 animals per group. Scale bars = 30 μm (**a**), 100 μm (**b-g,n-p**), 20 μm (**q,r**)

Combined immunostaining for neuronal markers NeuN and HuC/D in the cortical parenchyma of cytokine vector injected IFA only and IFA + MOG immunised animals, at both 28 and 56 dpi, showed large areas with some focal or diffuse loss of staining, extending from the pial surface to the deeper cortical layers, when compared to naïve animals (Fig. 4a-i). Co-staining with HuC/D and NeuN revealed a loss of Neun staining, or very weak staining, in some neurons that were still immunopositive for HuC/D (Fig. 4f-i), indicating the need to use the two markers to obtain accurate neuronal numbers. No neuronal loss was seen in GFP vector injected animals indicating that it was not the result of the surgical procedure (not shown). At both 28 and 56 days there was consistent loss of layer II neurons (illustrated at 56 days in Fig. 4b-e) along with reduced numbers in an expansive region spreading from the midline into layers V and VI above the cingulum bundle and corpus callosum (Fig. 4a,f-g). The extent of the neuronal loss in the midline regions was similar at 28 and 56 dpi and was not significantly different between IFA or MOG immunised animals (Fig. 4j). In cortical layers II/III there was a diffuse pattern of loss (Fig. 4b-d), with occasional discrete focal loss (Fig. 4e). The extent of this loss was not significantly different between IFA or MOG immunised and naïve animals at 28 dpi, but increased and became significant at 56 days (Fig. 4k). Decreased neuronal numbers were also found in the deeper cortical layer V (Fig. 4f-g), which was significant at 28 dpi for MOG immunised animals (24.8% reduction vs naïve) and at 56 dpi for both IFA (23.5% reduction vs naïve) and MOG immunised animals (28.7% reduction vs naïve) (Fig. 4l). The extent of neuronal loss was also quite extensive across the lateral surface of the cortex at 56 dpi for both IFA (14% reduction vs naïve) and MOG animals (24.6% reduction vs naïve) (Fig. 4m-o). Similar to the spread of demyelination, a loss of Neun/HuC-HuD staining was still detectable 1 mm anterior from the injection site (Fig. 4p). This reduction in neuronal number was accompanied by a decrease in the intensity of staining for both axonal 200 kDa neurofilament protein and dendritic MAP2 in the subpial cortical layers I-II (illustrated for cytokine vector injected MOG immunised animals at 28 dpi compared to the GFP vector control in Fig. 4q,r).

### Upregulation of genes related to apoptosis and necrosis in cortical tissue

Analysis of 86 genes related to TNF signalling and cell death in the cortical tissue showed an upregulation of over 40 genes related to apoptosis/necrosis and TNF cell death/cell survival signalling pathways in cytokine vector injected animals at 28 dpi when compared to GFP vector injected IFA or IFA/MOG controls (Table 2). The most significantly increased genes with a fold change of over 4 were TNFRSF14 (HVEM; 51.8 fold), NOX2 (Cytochrome B beta; 17 fold), TNFRSF1B (6 fold), DEFB1 (anti-viral defence; 5.9 fold), TNF (4.8 fold), TNFRSF5 (CD40; 4.8 fold), cIAP2 (4.3 fold), RIPK3 (4.2 fold), TNFRSF8 (CD30; 4.2 fold) and PYGL (4.1 fold). There was little difference in the pattern of gene expression between IFA or MOG immunised animals, although fold changes tended to be slightly greater in the MOG immunised animals.

**Table 2:** PCR array data showing the differential expression of cell death pathway genes in IFA aloned and MOG + IFA immunised animals injected with cytokine viral vectors compared to GFP vector injected control animals at 28 days post injection. Genes highlighted in bold and italics showed a greater than 2-fold significant increase

Gene	Fold change (compared to GFP control)					
	MOG			IFA		
	Fold Change	95% Cl	***p***-value	Fold Change	95% Cl	***p***-value
Aifm1	1.78	(1.28, 2.28)	0.017	***2.28***	(1.45, 3.10)	***0.033***
Ar	***3.32***	(1.47, 5.18)	***0.026***	***3.97***	(2.09, 5.85)	***0.008***
Atp6v1g2	***2.32***	(1.19, 3.45)	***0.035***	***2.81***	(1.92, 3.71)	***0.002***
Bax	1.45	(0.95, 1.96)	0.091	1.53	(1.02, 2.04)	0.054
Bid	1.94	(0.94, 2.95)	0.118	1.93	(1.53, 2.33)	0.002
Birc3	***4.33***	(2.12, 6.54)	***0.034***	***4.86***	(3.48, 6.23)3.48, 6.23)(1.47, 2.62)	***0.0001***
Bmf	2.23	(0.87, 3.59)	0.115	***2.05***	(1.47, 2.62)	***0.003***
Capn2	2.00	(1.31, 2.69)	0.053	***2.29***	(1.52, 3.05)	***0.013***
Capn3	***2.80***	(1.53, 4.06)	***0.042***	***2.20***	(1.42, 2.98)	***0.007***
Capn5	1.97	(1.66, 2.29)	***0.001***	1.97	(1.53, 2.41)	0.005
Capn7	1.80	(1.04, 2.55)	0.068	***2.07***	(1.57, 2.57)	***0.003***
Capns1	***2.53***	(1.44, 3.63)	***0.037***	***2.81***	(1.69, 3.92)	***0.021***
Cd40	***4.78***	(2.30, 7.25)	***0.047***	***3.91***	(2.59, 5.23)	***0.002***
Cetn1	***2.36***	(1.14, 3.59)	***0.033***	***2.09***	(1.04, 3.14)	***0.049***
Cyba	5.43	(2.06, 8.79)	0.107	***4.05***	(3.39, 4.71)	***0.0001***
Cybb	17.03	(3.33, 30.73)	0.059	***11.17***	(5.71, 16.64)	***0.003***
Cyld	***3.00***	(2.06, 3.93)	***0.007***	2.23	(1.06, 3.39)	0.070
Defb1	***5.87***	(3.01, 8.73)	***0.015***	***3.12***	(0.27, 5.97)	0.192
Dpys14	1.79	(1.32, 2.26)	***0.014***	***2.08***	(1.37, 2.79)	***0.027***
Fadd	1.71	(1.06, 2.35)	***0.041***	2.14	(1.19, 3.09)	0.066
Fas	3.49	(1.45, 5.54)	0.107	***2.24***	(1.77, 2.71)	***0.001***
Faslg	1.61	(0.88, 2.34)	0.167	***2.10***	(1.38, 2.81)	***0.014***
Foxi1	22.90	(0.00001, 65.05)	0.091	23.61	(0.00001, 72.42)	0.323
Glud1	2.06	(1.35, 2.78)	0.052	***2.14***	(1.36, 2.93)	***0.021***
Glul	***2.29***	(1.39, 3.18)	***0.021***	2.50	(0.95, 4.06)	0.088
Grb2	***2.65***	(1.95, 3.34)	***0.004***	***2.69***	(1.83, 3.55)	***0.007***
Hspbap1	***2.08***	(1.62, 2.55)	***0.005***	***2.22***	(1.45, 2.98)	***0.022***
Kcnip1	1.77	(1.03, 2.51)	0.050	***2.40***	(1.43, 3.36)	***0.019***
Madd	1.63	(1.03, 2.22)	0.046	***2.27***	(1.45, 3.10)	***0.022***
Myd88	2.91	(1.56, 4.27)	0.088	***2.52***	(1.99, 3.04)	***0.001***
Nfkb1	***2.34***	(1.69, 3.00)	***0.004***	***2.24***	(1.53, 2.95)	***0.007***
Ngf	***2.04***	(1.36, 2.27)	***0.008***	***2.37***	(1.39, 3.35)	***0.012***
Ngfr	***3.32***	(1.68, 4.96)	***0.024***	***2.59***	(1.28, 3.90)	***0.031***
Nox1	***3.44***	(1.77, 5.11)	***0.008***	***4.23***	(2.53, 5.94)	***0.001***
Parp1	1.85	(1.06, 2.64)	0.078	1.98	(1.48, 2.48)	0.004
Parp2	1.25	(0.90, 1.60)	0.200	1.36	(1.15, 1.57)	0.008
Ppid	***2.09***	(1.38, 2.80)	***0.036***	***2.23***	(1.59, 2.87)	***0.005***
Pygl	***4.06***	(1.84, 6.28)	***0.048***	***3.31***	(1.83, 4.79)	***0.010***
Rab25	***2.55***	(1.27, 3.83)	***0.027***	***2.42***	(1.45, 3.38)	***0.005***
Dennd4a	1.96	(1.25, 2.67)	0.041	***2.13***	(1.57, 2.70)	***0.003***
Ripk1	1.80	(1.29, 2.31)	0.022	1.94	(1.26, 2.62)	0.027
Ripk2	1.64	(1.17, 2.10)	0.056	1.88	(1.43, 2.34)	0.010
Ripk3	***4.16***	(1.83, 6.48)	***0.049***	***2.71***	(2.00, 3.42)	***0.0001***
Sp1	1.92	(1.31, 2.53)	0.036	***2.03***	(1.46, 2.61)	***0.012***
Spata2	***2.36***	(1.89, 2.82)	***0.0001***	***2.66***	(1.44, 3.88)	***0.038***
Tmem123	2.85	(1.60, 4.11)	0.065	***2.36***	(1.98, 2.75)	***0.000***
Tmem57	2.63	(1.12, 4.14)	0.055	***2.04***	(1.11, 2.97)	***0.018***
Tnf	***4.81***	(1.92, 7.71)	***0.049***	***3.38***	(1.69, 5.06)	***0.039***
Tnfrsf10b	***2.19***	(1.09, 3.29)	***0.037***	***2.23***	(1.32, 3.14)	***0.003***
Tnfrsf14	***51.83***	(0.00001, 120.6)	***0.028***	***34.17***	(0.00001, 79.86)	***0.030***
Tnfrsf17	***2.10***	(1.74, 2.47)	***0.001***	***2.15***	(1.58, 2.73)	***0.013***
Tnfrsf1a	***2.62***	(1.80, 3.44)	***0.018***	***2.21***	(1.55, 2.86)	***0.009***
Tnfrsf1b	***6.02***	(3.58, 8.46)	***0.014***	***4.12***	(3.36, 4.89)	***0.0001***
Tnfrsf25	1.91	(1.01, 2.80)	0.023	2.26	(0.78, 3.74)	0.130
Tnfrsf8	***4.24***	(2.84, 5.63)	***0.003***	***4.14***	(3.14, 5.15)	***0.0001***
Tnfrsf10	***2.63***	(1.57, 3.70)	***0.029***	***2.59***	(1.82, 3.37)	***0.001***
Tradd	***2.11***	(1.71, 2.52)	***0.002***	***2.10***	(1.46, 2.74)	***0.014***
Traf2	***2.30***	(1.60, 2.99)	***0.020***	***2.38***	(1.64, 3.11)	***0.009***
Txnl4b	2.91	(1.49, 4.32)	0.114	***2.23***	(1.62, 2.84)	***0.005***

### Expression of necrosis related proteins in cortical neurons

**Fig. 5:**
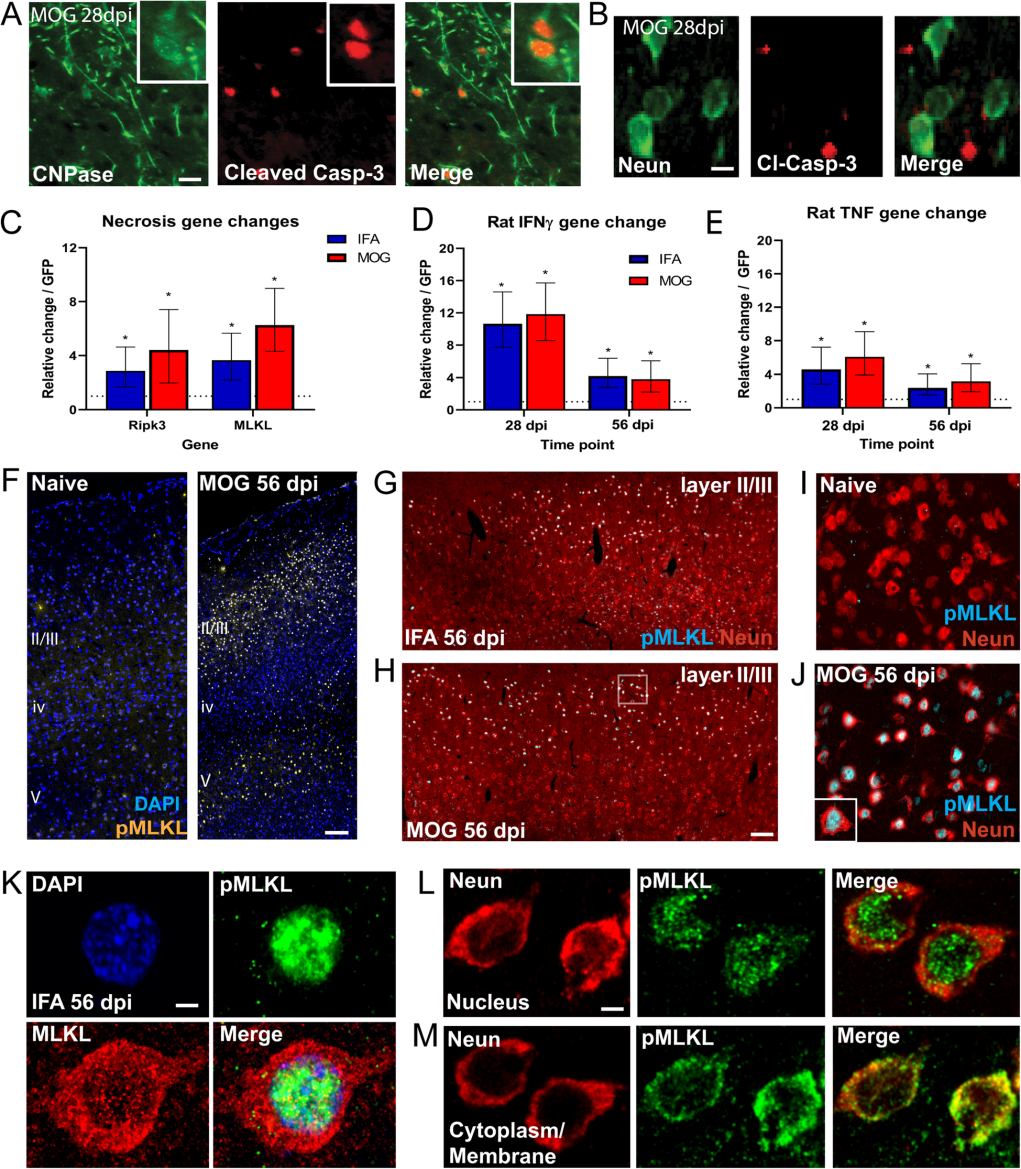
Expression of cell death related proteins in cortical neurons. CNPase+/Cleaved Casp-3+ oligodendrocytes are illustrated at 28 dpi in the cortical layers of MOG immunised animals injected with cytokine viral vectors, indicating apoptosis (**a**), whereas no NeuN+/Cleaved Casp-3+ neurons were found (**b**). QRT-PCR analysis showed increased gene expression for the necroptosis genes *RIPK3* and *MLKL* in cortical tissue from 28 dpi cytokine viral vector injected animals compared to GFP controls, with no significant difference between IFA or MOG immunised animals (**c**). Levels of endogenous rat IFNγ (**d**) and TNF (**e**) genes were upregulated at 28 and 56 dpi in both MOG and IFA immunised animals, with highest levels at 28dpi. Statistics C-E: t-tests on Ct values, *P < 0.05. Laminar distribution of pMLKL staining in naïve and MOG animals shows very minimal expression in naïve animals and significant expression in layers II/III and V in a MOG immunised animal at 56 dpi (**f**). This pattern was not different between MOG and IFA immunised animals (not shown). pMLKL expression was greatest in layers II-III in closest proximity to the subpial surface, with similar numbers of cells were present in IFA and MOG immunised animals (**g,h**). Co-staining for NeuN and pMLKL showed the absence of pMLKL in naïve cortex (**i**) and identified pMLKL-expressing cells as neurons in 56 dpi cytokine vector injected MOG immunised animals (**j**; from region in dashed box). Confocal imaging of neurons from the subpial cortical layers showed that pMLKL was upregulated in cytokine vector injected animals within the nucleus of neurons, whilst the unphosphorylated form was located in the cytoplasm (**k**). Although pMLKL was largely constrained to the nucleus in the majority of neurons (**k,l**), pMLKL could also be seen in the cytoplasm/membrane compartment in some neurons (**m**). Scale bars = 10 μm (**a,b**), 200 μm (**f-h**), 5 μm (**k-m**)

To investigate whether neurons and oligodendrocytes were dying via apoptosis, we used cleaved caspase-3 as a marker. Whilst we could detect cleaved caspase-3+/CNPase+ oligodendrocytes within the subpial layers where demyelination was present (Fig. 5a), no NeuN+/Cleaved caspase-3+ neurons were found (Fig. 5b). In contrast, there were significant increases in expression of the necroptosis related genes RIPK3 and MLKL within cortical tissue from both IFA only and IFA + MOG immunised animals injected with cytokine vectors, when compared to GFP vector injected rats (Fig. 5c), accompanied by increases in endogenous rat IFNγ and TNF gene expression within the cortical tissue at both 28 and 56 dpi in both animal groups (Fig. 5d,e). The final protein in the necroptosis pathway is MLKL, which in its phosphorylated form can produce oligomers able to form a pore in the cell membrane, leading to cell death. In naïve animals MLKL+ cells were rarely seen, whereas in cytokine vector injected animals there was a laminar distribution of pMLKL expression in cortical layers II/III and V with little staining in layer IV (illustrated for MOG immunised animals in Fig. 5f). This pattern of expression was not different between MOG and IFA immunised animals (not shown). pMLKL was expressed predominantly by cells in layer II/III, with greater expression in neurons in closest proximity to the subpial surface (Fig. 5g,h). Similar numbers of cells were present in IFA (Fig. 5g) and MOG immunised animals (Fig. 5h). The vast majority of these cells were NeuN+ neurons (Fig.5i,j), where pMLKL was expressed mainly in the nucleus. Within the subpial layers of the cortex, the unphosphorylated form of MLKL was largely constrained to the cytoplasm of neurons, whilst the phosphorylated form was seen within the nucleus (Fig. 5k). In a small proportion of pMLKL+ neurons located near regions of neuronal loss or in deeper cortical layer V, the pMLKL was expressed within the cytoplasm/membrane rather than the nucleus (Fig. 5l,m).

### Changes in gait by automated CatWalk analysis

**Fig. 6:**
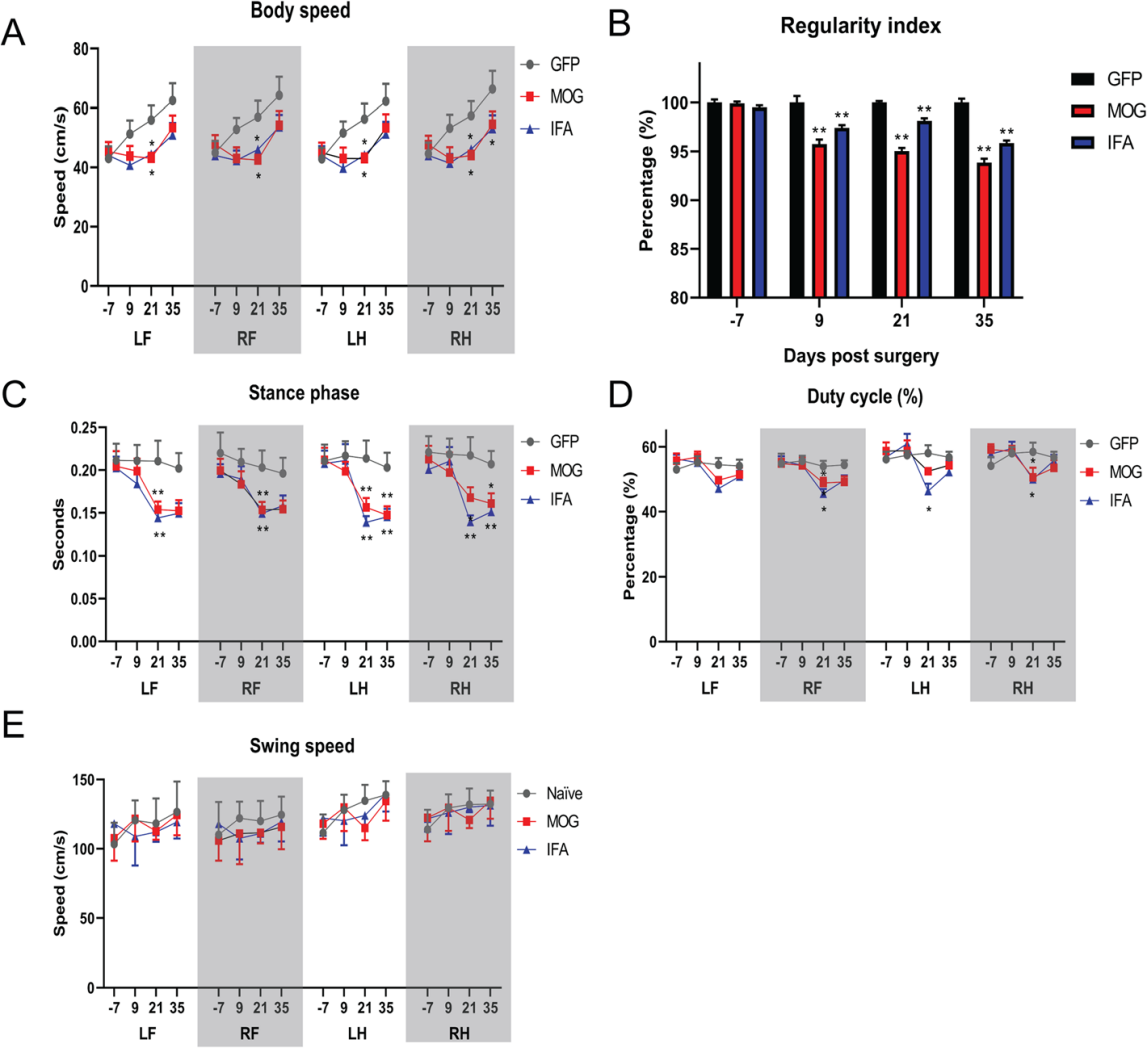
Changes to gait parameters measured using Catwalk XT. Following 1 week of training, a quantitative fully automated gait analysis was conducted on the animals 7 days before surgery and then repeated at 9, 21 and 35 days post injection of the cytokine or GFP lentiviral vector. The graphs show the average for each parameter from 6 compliant runs with separate lines for each of the paws (RF: right forelimb; LF: left forelimb; RH: right hindlimb; LH: left hindlimb). The body speed was significantly lower in both IFA and MOG immunised animals at 21 dpi compared to GFP (**a**). Both IFA and MOG animals showed a decrease in percentage of the regularity index (%), which is the number of normal step sequence patterns relative to the total number of paw placements used as a measure of interlimb coordination (**b**). Cytokine viral vector injection decreased the duration of the stance phase, which is the average time in seconds that the paw is in contact with the glass plate for each step cycle, of all paws at 21 dpi and the hind paws at 35 dpi (**c**). The duty cycle represents the percentage of time the paw accounts for the total step cycle of the paw and decreased for the right front and left and right hind paws at 21 dpi for IFA animals and at right front and hind paws for MOG animals in animals receiving cytokine viral vectors (**d**). There was no change to swing speed following cytokine injection at any timepoint (**e**). All values are given as mean ± SEM. Statistics: ANOVA with TUKEY post hoc test. *P < 0.01 compared to GFP injected group

The injection of the lentiviral vectors occurred into the sagittal sulcus at the level of the motor and sensory motor cortex. Therefore, we employed the CatWalk analysis to quantify any general effects on motor performance or control. Cytokine vector injected animals displayed a significant decrease in body speed for all paws at 21 dpi, when compared to GFP controls, that was not significantly different between MOG or IFA immunised animals (Fig. 6a). There was also a small significant decrease in the regularity index (Fig. 6b). We found a significant increase in the duration of the stance phase of all paws at day 21 dpi and the hind paws at day 35 (Fig. 6c). There was a decrease in duty cycle at 21 dpi for RF and RH in both MOG and IFA animals (Fig. 6d), but no change in swing speed of any paw (Fig. 6e).

## Discussion

The presence of immune cell infiltrates in the meninges is associated with lymphoid tissue development, greater cortical demyelination and significant neuronal and axonal loss in the progressive MS brain [15, 17, 22, 23, 30]. In this study, we provide experimental evidence that chronic production of TNF and IFNγ by meningeal cells in the cortical subarachnoid space gives rise to the persistent presence of meningeal immune cell infiltrates, subpial-demyelination and accumulating neuronal loss, similar to that seen in the MS cortex [23]. These data help to establish a causal relationship between the chronic production of cytokines in the meningeal compartment and cortical pathology in MS [24] and provide one possible mechanism for neuronal death through the TNF-induced necroptosis pathway.

Subarachnoid lentiviral vector injections were able to produce sustainable CSF TNF and IFNγ levels very similar to those reported for post mortem SPMS [14, 24]. By replicating at least part of the pro-inflammatory milieu seen in MS CSF [24], we were able to induce the formation of substantial immune cell infiltrates in the meninges that displayed similar cellular composition to meningeal inflammation reported in SPMS patients [22, 30], suggesting that CSF pro-inflammatory cytokine expression alone may be enough to induce the formation of meningeal lymphocyte aggregates in the subarachnoid space of the human MS brain [17, 22]. Whether the immune cell infiltrates seen in this study become organised to take on tertiary lymphoid organ structure and function and whether the nature of the infiltrates can be modulated by other cytokines will require further detailed study.

Sustained TNF and IFNγ expression produced subpial lesions that extended from the pial surface down to cortical layer III/IV and replicated key pathological characteristics of MS cortical type III lesions, including microglial activation, minimal lymphocytic infiltration and an absence of amoeboid macrophage-like cells [4, 17, 23, 28]. The subpial location and outside-in gradient of demyelination and microglial activation supports the hypothesis that a cytotoxic factor(s) diffusing from the meninges or CSF compartment contributes, either directly or indirectly, to GML pathogenesis [23, 29]. Indeed, although microglial activation was higher in MOG immunised animals, it was also present in IFA immunised animals in a pattern similar to SPMS cases with high meningeal inflammation [29], supporting the notion it can be driven by meningeal inflammation and was not a consequence of demyelination. IFNγ, a cytokine that is typically produced by Th1 and NK cells, can play a key role in activating the pro-inflammatory phenotype of microglia [2, 5], by inducing TNFR1 expression in microglia whilst inhibiting transcription of TNFR2 mRNA [36]. This would enhance the effect of soluble TNF from the CSF in promoting the pro-inflammatory cascade and result in the activation of neighbouring microglia located in deeper cortical layers. Constitutive TNFR1 expression is very low in the normal brain but is upregulated and occurs primarily on neurons and oligodendrocytes in the MS brain [25]. It has been shown to be induced in vitro by the presence of IFNγ [2, 36].

Previous studies have shown that acute injection of recombinant TNF and IFNγ protein into the cerebral subarachnoid space [14] or cortical grey matter parenchyma [26] produced subpial demyelination that rapidly resolved and required a pre-existing autoimmune anti-MOG immune, as no demyelination occured in IFA only immunised animals. In the current chronic model, IFA immunised animals developed subpial lesions, that were devoid of T- and B-cells, and loss of olig2+ cells by 2 months post cytokine vector injection, demonstrating that chronic exposure of oligodendrocytes to these cytokines will eventually lead to cell death and demyelination that is not due to infiltration of cytotoxic immune cells into the parenchyma or circulating autoantibody expression. Indeed, this is in keeping with in vitro studies on rat oligodendrocyte progenitors showing that IFNγ can regulate TNFR1 expression and act synergistically with TNF to potentiate TNF signalling and increase cell death [1]. Whilst an anti-MOG response and involvement of the complement system [39] may increase the speed and severity of MS-like pathology, indicated by the more extensive demyelination in MOG immunised animals, it does not appear to be necessary for the development of chronic meningeal inflammation and accumulating neuronal loss, which were not significantly different between the two animal groups. In this respect it is of interest to note that non-MS inflammatory CNS conditions that are accompanied by meningeal inflammation, such as viral meningitis and tuberculosis meningitis, are not characterised by extensive subpial demyelination [13, 19, 22], despite the likely presence of elevated pro-inflammatory cytokines in the CSF. This points towards an MS-specific mechanism underlying the subpial demyelination, which agrees with the finding that an anti-myelin immune response is necessary for very extensive demyelination in our current model. The possible contribution of intrathecal anti-myelin antibodies to subpial cortical demyelination in MS patients has yet to be fully addressed. However, it is not known whether neuronal loss is also a characteristic of the non-MS inflammatory CNS conditions.

Grey matter neuronal loss in the cerebral cortex, that is independent of demyelination, is a reproducible finding in the progressive MS brain and is relatively widespread [7, 10, 15, 23, 41]. It occurs in a decreasing gradient away from the pial surface in SPMS cases that have meningeal inflammation and lymphoid tissue formation [23]. It is suggested that it has an inflammatory origin but this has hitherto remained untested. In the current study, we observed a similar pattern of neuronal loss, which was greatest and occurred at an increased rate in midline regions that were in close proximity to the dense immune cell infiltrates. Significant neuronal loss was not seen in subpial regions away from the midline until longer periods after viral vector injection. This may suggest that slowly accumulating damage to individual neurons eventually leads to cell death. Neuronal loss has been previously observed at 30 days after TNF and IFNγ infusion directly into the cortex, but was suggested to be a temporary impairment of NeuN expression rather than actual loss of neurons, as a recovery of neuronal numbers was seen [34]. We also observed loss of NeuN expression despite the continued presence of the neuron, so to overcome this issue we used combined immunostaining of neurons with Neun and HuC/D to reduce the chances of a loss of protein expression rather than cellular loss. In contrast to this earlier study, we observed an accumulating neuronal loss over time with no recovery between 28 and 56 days, which is what might be expected due to persistent exposure to a cytotoxic stimulus. Previous research on MS cortical pathology using animal models has been limited by the fact that the experimental paradigm has involved direct cortical injection resulting in significant cortical damage [26, 34]. Injection into the subarachnoid space, to replicate the situation in the MS meninges and brain, caused very minimal damage to the blood brain barrier or surrounding tissue, as demonstrated by the lack of pathology in GFP vector injected animals. In addition, neuronal loss occurred in the lateral cortex and in subpial layers up to 1 mm away from the injection site making it extremely unlikely that it was a direct result of tissue trauma. As neuronal loss could be identified in regions that showed minimal loss of MOG staining and in both IFA and MOG animals, this would suggest that neuronal loss occurred independently of grey matter demyelination, similar to the situation seen in the MS cortical grey matter [7, 15, 23, 28].

Despite neuronal loss being a hallmark feature of GM pathology, apoptotic caspase-3+ neurons are very rare in human MS cortical layers [23, 28], suggesting apoptosis may not be the key mechanism of neuronal cell death in MS GM. Instead, as in many chronic inflammatory conditions outside the CNS [9, 11, 16], soluble TNF may be acting via TNFR1 receptors on neurons to activate necroptosis. After assembly of the death receptor complex IIb, necroptosis involves the phosphorylation of RIPK1 and RIPK3, followed by recruitment and phosphorylation of the mixed lineage kinase domain-like (MLKL) pseudokinase, which then oligomerises before migration to the cell membrane to cause cell membrane disruption and ultimately cell death [35, 37]. In keeping with this, our recent gene expression profiling study of post-mortem MS cortical grey matter demonstrated a shift in the balance of TNF signalling towards the TNFR1 and RIPK3/MLKL pro-necroptotic pathway in the presence of lymphoid-like meningeal inflammation [25]. In support of this human tissue data, the genes for RIPK3 and MLKL were upregulated in the cortex following cytokine vector injection, which was accompanied by an increase in immunostaining for pRIPK3 and MLKL specifically in neurons, where the phosphorylated form of MLKL was localised both in the nucleus and cytoplasmic compartment. The nuclear localisation is in keeping with recent findings demonstrating a relocation of MLKL and RIPK3 to the nucleus prior to phosphorylation [40]. Although the TNFR2 receptor, which is known to mediate predominantly neuroprotective responses [33], was significantly upregulated in cortical GM in the current study, the presence of neuronal loss and the upregulation of necroptosis signalling suggests that the majority of TNF signalling was occuring via the TNFR1 dependent necroptotic pathway. RIPK3 mediated necroptosis in oligodendrocytes has been shown to occur in MS white matter lesions [27], although this did not appear to be the case in the cortical grey matter in our model. Further work is needed to examine the detailed molecular cascades in human MS grey matter to understand how meningeal inflammation may be related to activation of this pathway and at which point it becomes irreversible.

Although an extensive behavioural analysis was not carried out, the automated catwalk analysis detected subtle changes in gait in animals that had received cytokine vector injections. In particular, a decrease in the regularity index may demonstrate that cytokine induced subpial injury to the motor cortex can affect coordination between the limbs. The increase in stance phase also seen here is in agreement to previous reports which found damage to the motor cortex can cause reduced weight bearing [32, 38]. These effects on gait are also very similar to those reported for models of neuropathic pain [18], which is also a common symptom in MS patients that develops secondary to demyelination and neuroinflammation and axonal damage [20].

In conclusion, we have developed an experimental model of chronic cortical pathology, that can be induced in a targeted manner to a defined anatomical location and recapitulates many of the pathogenic mechanisms in MS including meningeal inflammation, demyelination and neuronal loss. This model has allowed us to demonstrate the possible involvement of TNF mediated necroptosis as a mechanism of neuronal death that most likely contributes to the complex pathology that characterises the progressive stages of MS. Although the current results cannot distinguish between a direct or indirect role for TNF and IFNγ in inducing cortical pathology, they do show that the persistent elevation of the CSF levels of these two cytokines could be a trigger. The detailed molecular mechanisms involved in grey matter tissue damage in MS can now be further tested through the use of therapeutic interventions using this experimental approach to determine whether TNF signalling may be playing a direct and/or indirect role.

## Supplementary information

**Additional file 1.** Generation and characterisation of lentiviral vectors for overexpression of TNF and IFNγ in rat meningeal cells. Schematic drawing of the plasmids used for production of VSVg pseudotyped LVs (A). Vectors were generated by 4-plasmid co-transfection using the pMD2-LgRRE and pRSV-rev packaging plasmids, VSVg envelope plasmid and pRRLsincppt-CMV-TNF/IFNγ-WPRE genome plasmid in Hek293T cells. Human codon optimised TNF or IFNγ sequences were cloned into pRRL-sinccpt-CMV-WPRE HIV-1 genome plasmid under the control of an optimised human cytomegalovirus (CMV) promoter. Rat primary meningeal cells were transduced at MOI 50 with LVs expressing either eGFP, TNF or IFNy. Cells were stained with antibodies specific to human TNF or IFNγ protein 72 h after transduction (B: scale bar 5 μm). Human TNF and IFNγ levels in cell supernatants from rat primary meningeal cells transduced with LVs expressing either TNF or IFNy lentivirus at MOI 100 were measured at 24 and 48 h by enzyme-linked immunosorbent assay for human TNF or IFNγ protein (C).

**Additional file 2.** Quantification of anti-MOG antibody titres in MOG immunised rats. Peripheral anti-MOG titres were quantified in serum from terminal blood samples taken at 28 dpi from rats injected with 5μg of rmMOG: for (A) total IgG, (B) IgG1, and (C) IgG2a. All data is presented as mean ± SEM.

**Additional file 3.** Immunostaining for CNPase at 56 dpi in MOG immunised animals injected with GFP (A) or cytokine (B) viral vectors to confirm demyelination. Scale bar = 300 μm.

## Data Availability

The dataset(s) supporting the conclusions of this article are included within the article and its additional files.
