# Supplementary figures and images for: Persistent elevation of intrathecal pro-inflammatory cytokines leads to multiple sclerosis-like cortical demyelination and neurodegeneration
Source: Acta Neuropathol Commun. 2020 May 12;8:66. doi: 10.1186/s40478-020-00938-1 (PMC7218553; doi:10.1186/s40478-020-00938-1)

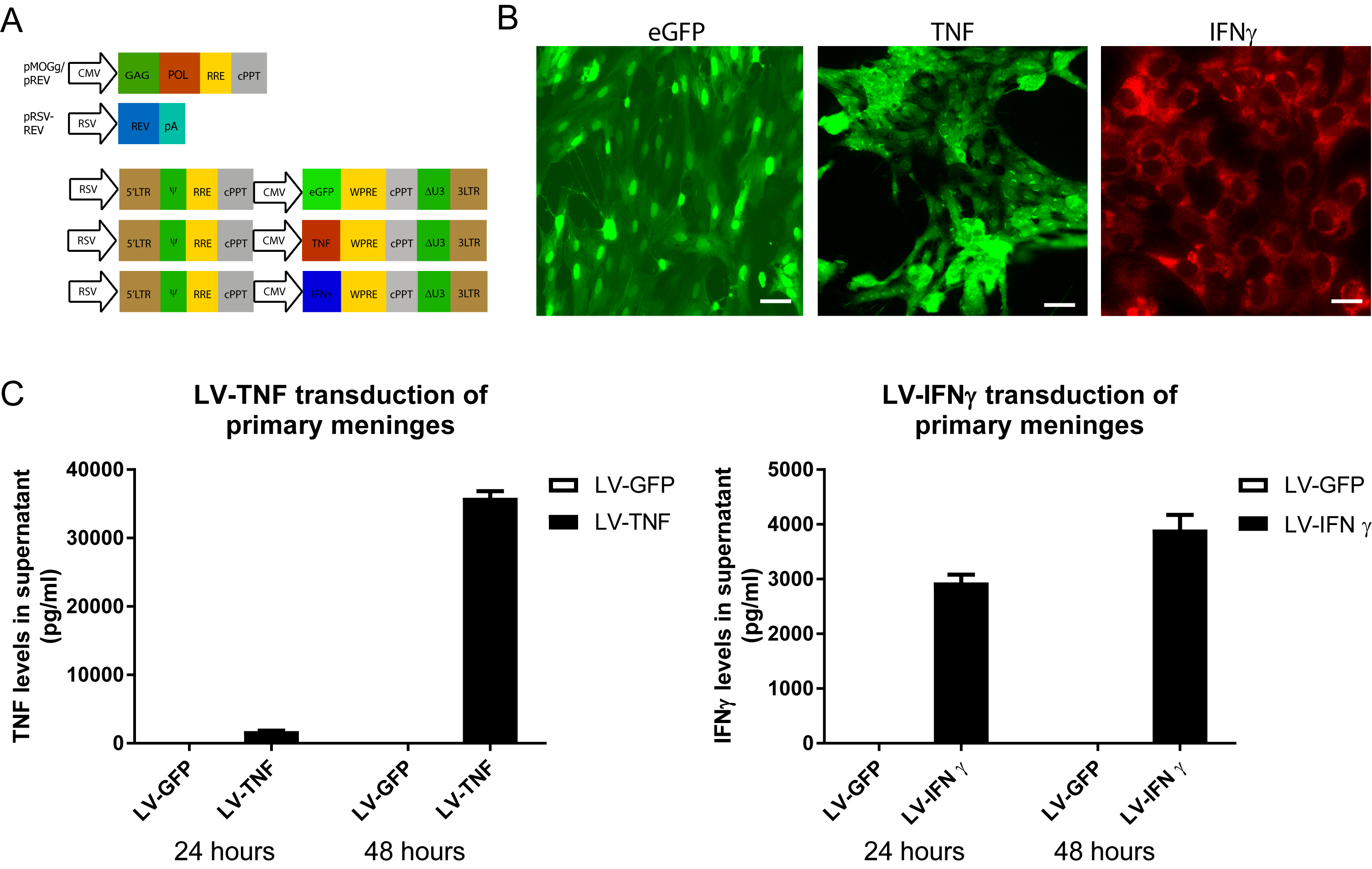

Supplement: Supplementary file 1 — Additional file 1. Generation and characterisation of lentiviral vectors for overexpression of TNF and IFNγ in rat meningeal cells. Schematic drawing of the plasmids used for production of VSVg pseudotyped LVs (A). Vectors were generated by 4-plasmid co-transfection using the pMD2-LgRRE and pRSV-rev packaging plasmids, VSVg envelope plasmid and pRRLsincppt-CMV-TNF/IFNγ-WPRE genome plasmid in Hek293T cells. Human codon optimised TNF or IFNγ sequences were cloned into pRRL-sinccpt-CMV-WPRE HIV-1 genome plasmid under the control of an optimised human cytomegalovirus (CMV) promoter. Rat primary meningeal cells were transduced at MOI 50 with LVs expressing either eGFP, TNF or IFNy. Cells were stained with antibodies specific to human TNF or IFNγ protein 72 h after transduction (B: scale bar 5 μm). Human TNF and IFNγ levels in cell supernatants from rat primary meningeal cells transduced with LVs expressing either TNF or IFNy lentivirus at MOI 100 were measured at 24 and 48 h by enzyme-linked immunosorbent assay for human TNF or IFNγ protein (C). [file 40478_2020_938_MOESM1_ESM.tif]

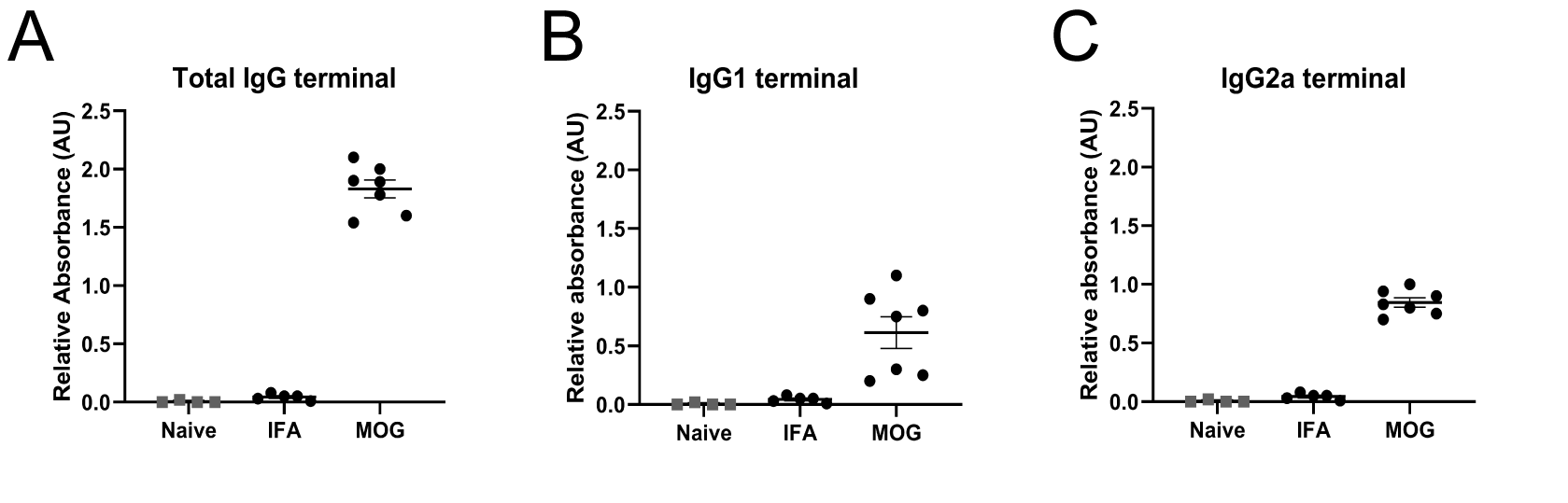

Supplement: Supplementary file 2 — Additional file 2. Quantification of anti-MOG antibody titres in MOG immunised rats. Peripheral anti-MOG titres were quantified in serum from terminal blood samples taken at 28 dpi from rats injected with 5μg of rmMOG: for (A) total IgG, (B) IgG1, and (C) IgG2a. All data is presented as mean ± SEM. [file 40478_2020_938_MOESM2_ESM.tif]

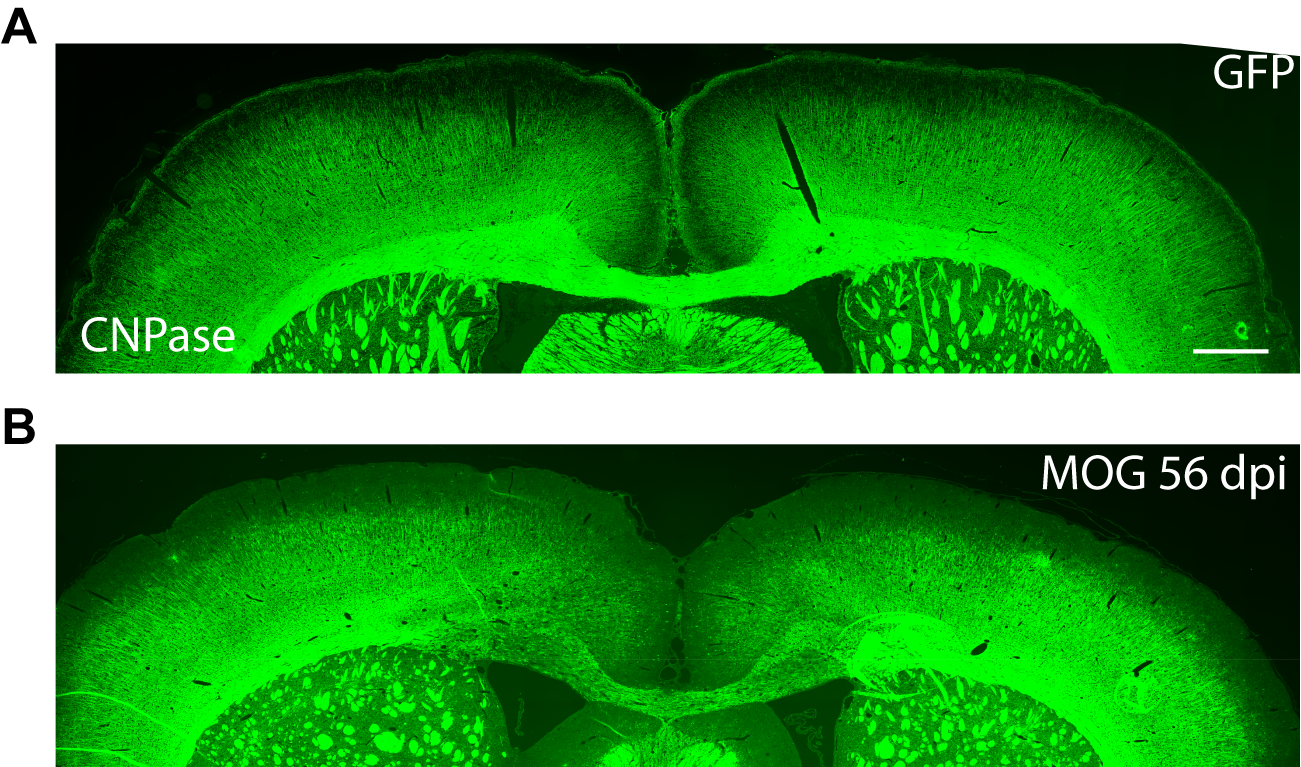

Supplement: Supplementary file 3 — Additional file 3. Immunostaining for CNPase at 56 dpi in MOG immunised animals injected with GFP (A) or cytokine (B) viral vectors to confirm demyelination. Scale bar = 300 μm. [file 40478_2020_938_MOESM3_ESM.tif]
